# Large-scale impacts of sea star wasting disease (SSWD) on intertidal sea stars and implications for recovery

**DOI:** 10.1371/journal.pone.0192870

**Published:** 2018-03-20

**Authors:** C. Melissa Miner, Jennifer L. Burnaford, Richard F. Ambrose, Liam Antrim, Heath Bohlmann, Carol A. Blanchette, John M. Engle, Steven C. Fradkin, Rani Gaddam, Christopher D. G. Harley, Benjamin G. Miner, Steven N. Murray, Jayson R. Smith, Stephen G. Whitaker, Peter T. Raimondi

**Affiliations:** 1 Department of Ecology and Evolutionary Biology, University of California, Santa Cruz, California, United States of America; 2 Department of Biological Science, California State University, Fullerton, California, United States of America; 3 Department of Environmental Health Sciences, University of California, Los Angeles, California, United States of America; 4 Olympic Coast National Marine Sanctuary, Port Angeles, Washington, United States of America; 5 Padilla Bay National Estuarine Research Reserve, Padilla Bay, Washington, United States of America; 6 Valentine Eastern Sierra Reserves, University of California, Santa Barbara, California, United States of America; 7 Marine Science Institute, University of California, Santa Barbara, California, United States of America; 8 Olympic National Park, Port Angeles, Washington, United States of America; 9 Department of Zoology, University of British Columbia, Vancouver, British Columbia, Canada; 10 Institute for the Oceans and Fisheries, University of British Columbia, Vancouver, British Columbia, Canada; 11 Department of Biology, Western Washington University, Bellingham, Washington, United States of America; 12 Department of Biological Sciences, California State Polytechnic University, Pomona, California, United States of America; 13 Channel Islands National Park, Ventura, California, United States of America; Department of Agriculture and Water Resources, AUSTRALIA

## Abstract

Disease outbreaks can have substantial impacts on wild populations, but the often patchy or anecdotal evidence of these impacts impedes our ability to understand outbreak dynamics. Recently however, a severe disease outbreak occurred in a group of very well-studied organisms–sea stars along the west coast of North America. We analyzed nearly two decades of data from a coordinated monitoring effort at 88 sites ranging from southern British Columbia to San Diego, California along with 2 sites near Sitka, Alaska to better understand the effects of sea star wasting disease (SSWD) on the keystone intertidal predator, *Pisaster ochraceus*. Quantitative surveys revealed unprecedented declines of *P*. *ochraceus* in 2014 and 2015 across nearly the entire geographic range of the species. The intensity of the impact of SSWD was not uniform across the affected area, with proportionally greater population declines in the southern regions relative to the north. The degree of population decline was unrelated to pre-outbreak *P*. *ochraceus* density, although these factors have been linked in other well-documented disease events. While elevated seawater temperatures were not broadly linked to the initial emergence of SSWD, anomalously high seawater temperatures in 2014 and 2015 might have exacerbated the disease’s impact. Both before and after the onset of the SSWD outbreak, we documented higher recruitment of *P*. *ochraceus* in the north than in the south, and while some juveniles are surviving (as evidenced by transition of recruitment pulses to larger size classes), post-SSWD survivorship is lower than during pre-SSWD periods. In hindsight, our data suggest that the SSWD event defied prediction based on two factors found to be important in other marine disease events, sea water temperature and population density, and illustrate the importance of surveillance of natural populations as one element of an integrated approach to marine disease ecology. Low levels of SSWD-symptomatic sea stars are still present throughout the impacted range, thus the outlook for population recovery is uncertain.

## Introduction

Infectious diseases are prevalent in terrestrial and marine systems (reviewed in [1–3]), and can result in mass mortality of a species or suite of species. Evidence suggests that disease outbreaks in marine systems are becoming increasingly common, potentially as a consequence of the shift in environmental conditions associated with global climate change [2,4]. When a disease outbreak affects a foundation species [5], ecosystem engineer [6] or keystone species [7], disease-induced changes in abundance, size structure, and individual behavior and performance can ripple through the entire ecosystem, causing substantial and long-term changes in system structure and function. Examples of ‘marine disease emergencies’ [8] in which ecologically critical species have been affected by disease outbreaks and subsequent mass mortalities include eelgrass wasting disease (reviewed in [9]), white-band disease in reef-building corals [10], an unidentified pathogen affecting the tropical urchin *Diadema antillarum* [11] and, as reported here, sea star wasting disease (SSWD) in the ochre sea star *Pisaster ochraceus*.

SSWD is an ongoing disease epidemic, which has devastated intertidal and nearshore sea star populations along much of the west coast of North America [8,12–15]. *P*. *ochraceus* is one of the most recognized species from the intertidal and shallow subtidal zones along this coast, with a broad geographic range from Prince William Sound Alaska, USA to Cedros Island, Baja California, Mexico [16]. It is a generalist predator, with a diet that includes both sessile and mobile invertebrate prey items from multiple taxa (including annelid worms, barnacles, brachiopods, chitons, mussels, and whelks: [7,17–18]). *P*. *ochraceus* was the first species to be termed a ‘keystone predator’ [7] because of its strong effects on diversity of primary space occupiers in wave-exposed areas [7,19]. Even in areas where *P*. *ochraceus* does not play a keystone role, it is still a dominant predator [17,19] and thus is considered an ecologically important species throughout its range.

Determining the long-term effects of SSWD on *P*. *ochraceus* populations and on rocky intertidal communities poses a particular challenge for ecologists because the etiology of the disease is not yet fully resolved (although see [12] for identification of an associated densovirus). Early signs of SSWD include a twisted or deflated appearance, followed by the development of lesions (authors’ pers. obs); [12–14,20]. Because these early symptoms are similar to those resulting from other sources of stress in sea stars, such as desiccation or injury from predators (authors’ pers. obs.), it is impossible to know for sure that SSWD is present in a population until large numbers of animals enter the later stages of the disease (identified by large-scale tissue degradation, body fragmentation, authors’ pers. obs; [12–14, 20]) or die. The progression of visible signs of the disease can be rapid, on the scale of days. In fact, this short time-frame has led some pathologists to argue that “wasting” disease is a misnomer, as this term suggests a gradual reduction in body mass (M.M. Garner pers. com.). After SSWD-induced mortality, decomposition occurs quickly. Although observers in low-flow subtidal systems have noted piles of disarticulated ossicles (skeletal elements) as indicators of recent SSWD-induced sea star mortality, water motion in the intertidal zone quickly disperses decomposed bodies of diseased asteroids after death. Because of this combination of factors, monitoring efforts initiated at the start of the disease outbreak were unlikely to provide accurate characterizations of changes in *P*. *ochraceus* populations at any given location. Without “pre-outbreak” data to provide historical context, it is impossible to evaluate the impact of the disease or the trajectory of recovery for a *P*. *ochraceus* population. In these circumstances, data from ongoing monitoring programs provide the best means of assessing the impact of this disease.

This is not the first documented disease outbreak in asteroids on the North American Pacific coast; since the 1970s, outbreaks of (uncharacterized) diseases have been documented in southern California [21], the Gulf of California [22], and British Columbia [23]. Data from these prior disease events indicate that most outbreaks were spatially and temporally limited. For example, in 1997 diseased individuals were reported only as far south as Punta Banda, Baja California and as far north as Punto Estero California, spanning approximately 4 degrees of latitude [24]. The current epidemic is strikingly different from previous events in both geographic extent and persistence, and has contributed to the growing push for the development of coordinated responses to marine diseases (e.g., [8,25–27]). An underlying goal of this coordinated effort would be to develop better forecasting tools for disease events. With a vast number of conditions and stressors that could be associated with any disease outbreak, identifying specific ‘warning signs’ and generating predictions for disease onset or impact are not simple tasks [4]. Yet among these many possibilities, two factors stand out with strong potential for predicting disease affecting sea stars: temperature and population density. Both have been linked to the emergence and severity of prior disease events in marine systems (reviewed in [2,4]) and are widely considered to be important in the development of predictive models [1,2,4,27–29].

Once a disease outbreak does occur, especially one associated with a mass mortality event [30], the ability to assess the degree of population recovery becomes critically important. Historical data provided by long-term population surveillance are essential at this stage, as they allow the construction of ‘benchmark’ indices based on “normal” pre-disease population parameters, such as size frequency distributions and abundance. A variety of components factor into population recovery, including recruitment of new individuals, juvenile survivorship to the adult stage, and disease persistence. Each of these components can vary among affected populations, thus it is necessary to have long-term coordinated monitoring of multiple populations in order to: a) build recovery benchmark targets based on specific data, b) make informed assessments of recovery at various scales (e.g. within and among regions), and c) compare recovery estimates at various scales in order to identify factors that might facilitate or hinder recovery.

Here we have combined data from several long-term monitoring programs from mainland and island sites along the North American Pacific coast, ranging from Sitka, Alaska to San Diego, California. Most of these surveys were completed by partners in the Multi-Agency Rocky Intertidal Network (MARINe), who have been working with a standardized set of monitoring protocols to study community dynamics in rocky intertidal systems for up to 25 years. This unique data set provides the necessary temporal context to allow us to assess *P*. *ochraceus* population changes, and expansive spatial coverage to allow us to evaluate the impact of the disease across nearly the entire species’ range. We use these data to: 1) present regional patterns of difference in the degree to which SSWD has impacted *P*. *ochraceus* populations, 2) examine whether sea star density might have played a role in disease severity, 3) explore the potential relationship between *P*. *ochraceus* decline and water temperature, which has been implicated as a contributing factor in prior wasting events, and 4) present spatial patterns in *P*. *ochraceus* recruitment and juvenile survival between pre-and post- outbreak periods that can be used to 5) assess the potential for recovery of *P*. *ochraceus* populations within SSWD-impacted areas.

## Materials and methods

### Ethics statement

No animals were collected for these surveys, but many sites required permits for general research or permission for access. In Washington, work at Post Point was conducted under Washington Department of Fish and Wildlife (DFW) permit # 120720–1 and access to sites on Makah land and the Quinault Nation was granted to Olympic Coast National Marine Sanctuary (NMS) for all surveys. In Oregon, surveys were completed under Oregon DFW permit #’s 18084, 18610, 19306, 20174, and 21411, and access to Fogarty Creek was provided through collaborators at Oregon State University, who have an agreement with the owners. In California, the National Oceanic and Atmospheric Administration Office of National Marine Sanctuaries Program permitted surveys at sites within the Monterey Bay and Gulf of the Farallones NMS (MBNMS-2010-001-A1, MBNMS-2015-015-A1, and MULTI-2017-010-A1). Work at other California sites (including Marine Protected Areas) was authorized by California DFW permits SC-4055, SC-3124, SC-8187, SC-10589, and SC-003922. Annual permits to work at sites located in California State Parks were granted by the California Department of Parks and Recreation as follows: Channel Coast District State Parks to R. Ambrose and S. Lee, Crystal Cove State Park to S. Murray, J. Smith, and J. Burnaford, and State Park system-wide permits to P. Raimondi. Permission to work at Bodega, Coal Oil Point, Scripps Reef, and all sites on Santa Cruz Island was granted by the UC Natural Reserve System and the Nature Conservancy. The Sea Ranch Association allowed access to our site there. An access agreement was granted annually to the PISCO group at UCSC from the Pebble Beach Company to do surveys at Stillwater Cove. Hopkins Marine Station provided access to the site within their reserve. El Sur Ranch allowed access to Andrew Molera. Permission to access sites located on Vandenberg Air Force Base was granted by the U.S. Air Force. Access to Government Point was provided by Bixby Management Inc. Access to Alegria was approved annually by the Hollister Ranch Corporation. Sites not specifically listed above as requiring permits were either on public land or were surveyed by the organization in charge of granting access or permits (e.g., National Park Service).

### Study sites

**Fig 1:**
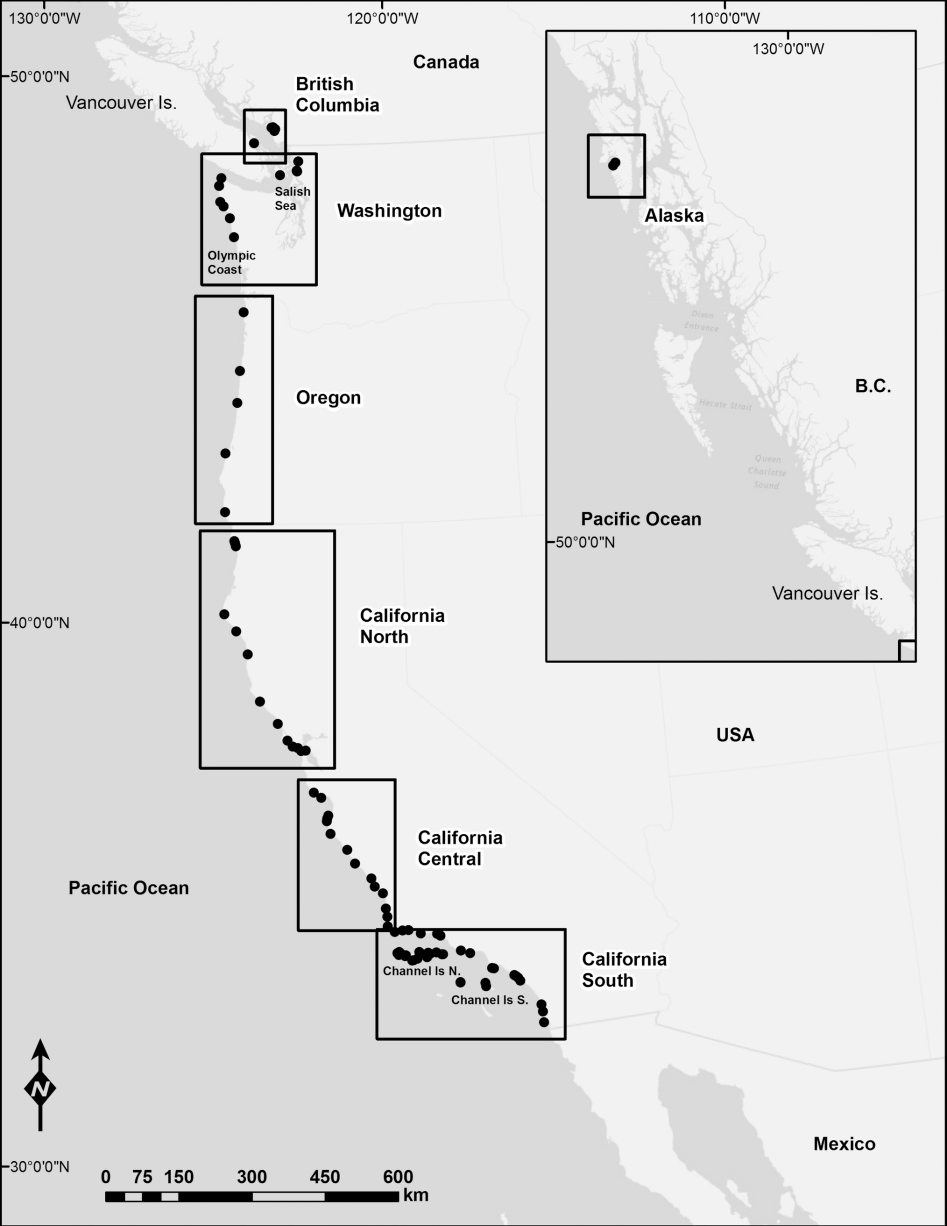
Map of study sites along the Pacific coast of North America. Sites are indicated by dots and study regions by labeled boxes. Refer to S1A–S1H Fig for more detail.

MARINe is a consortium of 18 groups (including state, federal, university, and private organizations) that conduct coordinated annual monitoring of intertidal community parameters at over 130 sites in four US states. By combining data from MARINe groups with data from the University of British Columbia, we present data on populations of *P*. *ochraceus* at 90 rocky intertidal sites spanning the North American Pacific coast, from southeast Alaska to San Diego County, California (Fig 1, S1A–S1H Fig, S1 Table). Physical site characteristics such as rock type, bench slope and size, wave exposure, temperature (both air and water), and sand influence vary substantially across this broad stretch of coastline, but all sites contained appropriate habitat for *P*. *ochraceus*. Detailed site descriptions, including monitoring duration and frequency, photos, and trends for other species monitored, can be found at pacificrockyintertidal.org.

### Abundance and size structure: Long-term monitoring

*P*. *ochraceus* were counted, measured, and (beginning in 2013) assigned to disease categories annually or semiannually at 90 long-term monitoring (LTM) sites in Alaska, Washington, Oregon, and California. At LTM sites where abundance of sea stars was initially high, three irregularly-shaped plots (20 to 160 m^2^) were established in areas of high *P*. *ochraceus* density, where individuals of all post-settlement life stages could be found; the exact configuration and plot size at a given site were dependent on available habitat and sea star density. At LTM sites with low densities of *P*. *ochraceus*, timed searches or “whole-site” searches were conducted within boundaries delineated by permanent marker bolts or GPS coordinates. At LTM sites in British Columbia, Canada, whole-site counts (no sizes) of *P*. *ochraceus* were made within fixed spatial boundaries from 2008 to 2016.

At LTM sites where *P*. *ochraceus* individuals were sized, the “radius” of each *P*. *ochraceus* encountered within the defined search area was measured as the distance from the middle of the star to the tip of the longest ray. Measurements were estimated to the nearest 10 mm for all stars with a radius larger than 7 mm. Individuals with a radius between 3 and 7 mm were recorded in the ‘5 mm’ radius bin. We did not regularly record stars with a radius below 3 mm as these cannot be reliably detected across large search areas with the naked eye.

Monitoring of *P*. *ochraceus* began as early as 1989 at some sites, but prior to 2000, stars were counted but not sized. Thus, only data collected in 2000–2016 are presented here. Because these data are collected on an annual (or semiannual) basis, they are well-suited for examining long-term trends in sea star abundance and population size structure across a large geographic area. Most LTM sites south of San Francisco, CA were sampled semiannually each spring and autumn, while most sites to the north were sampled annually in the summer except for those in British Columbia, which were monitored in the winter. After SSWD was first detected, we added a standardized disease monitoring protocol to all LTM surveys. Starting in late summer / fall 2013, each encountered star was assigned a disease category based on the 0–4 scale developed by Bates et al. [23], with a designation of ‘0’ indicating a state of “no visible disease–presumably healthy” and ‘4’ indicating “severe wasting”. Surveys done in 2000–2013 are considered “pre-onset of SSWD” or “pre”, and 2014–2016 are considered “post-onset of SSWD” or “post”.

### Density estimates: Coastal biodiversity surveys

Differences in plot size, tidal height, and habitat features among LTM sites complicate conversion of permanent plot counts to population density estimates, and thus preclude direct among-site comparisons of abundance from data collected using LTM methods. Instead, we used pre-onset of SSWD data from the Coastal Biodiversity Survey (CBS) Program [31] to compare *P*. *ochraceus* population densities among 68 sites across the US west coast where we also have LTM data that could be used to assess impact due to SSWD (described above). In the CBS protocol, *P*. *ochraceus* were counted along 2 meter-wide vertical swaths, which extended from the very high intertidal zone to the very low intertidal zone (to the lowest emersed point during low tides). Only the mid to low zone contains habitat appropriate for *P*. *ochraceus*, thus when area sampled was calculated for each site, we subtracted the high zone portion of the swaths beyond which no *P*. *ochraceus* occurred. Area searched ranged from 52 m^2^ to 3585 m^2^ among sites depending on the number of transects surveyed (typically 11) and the intertidal bench width. Only stars ≥ 50 mm diameter were recorded in CBS surveys, so they capture adult densities well, but exclude juveniles. CBS surveys were conducted at approximately 3–5 year intervals; at sites where multiple surveys had been done prior to 2013, the mean pre-SSWD density was used. To determine whether sites with higher densities of sea stars were more likely to experience population decline due to SSWD, and whether a potential relationship differed among regions, we ran an ANCOVA analysis assessing the severity of decline (# of stars counted in 2015 in LTM plots / long-term mean # of stars counted in LTM plots pre-SSWD) as a function of *P*. *ochraceus* density (from CBS swath data), region, and the interaction between density and region.

### Sea surface temperature

Intertidal temperature data have been continuously recorded, typically at 15-minute intervals, at select sites from Washington to Point Conception, California, beginning as early as 1999 (with different start years for different sites). Because of changes in temperature logger technology, three different data loggers (all from Onset Computer Corporation) have been used over time: HOBO pendant UA-002-64, HOBO UTBI-001 TidbiT, and HOBO UTBI-001 TidbiT v2. Loggers at most sites were housed in stainless steel wire mesh cages and bolted to the substrate. At some Washington sites, loggers were either encased in epoxy for protection and then bolted and epoxied to the bedrock, or housed in flow-through PVC tubes that were bolted to the substrate. Temperature loggers were installed in the mid-low intertidal zone, in areas that would afford some protection from waves and theft. No attempt was made to standardize factors that could affect temperature during emersion, such as shading, angle of incidence to sun, etc. Therefore, we used temperature data only from periods when loggers were fully submerged and recording seawater temperature. Because intertidal temperature loggers were not installed at sites in southern California, temperature data from four piers in the region [32] were used as a proxy. These piers are located in protected sandy areas, which can have elevated temperatures as compared to rocky shores (Raimondi per. obs.). However, because all temperature data are presented as deviations from the long-term mean for a given date rather than actual temperatures, we assumed patterns to be representative of the region. We did not formally test the relationship between water temperature and sea star declines because the temporal scale of our population surveys was too coarse to capture the temporal scale at which *P*. *ochraceus* responds to its environment (e.g., [33]).

### Juvenile abundance and impact of SSWD on juvenile survivorship

Following the convention established by Feder [34] and Sewell and Watson [35], we consider ‘juvenile’ *P*. *ochraceus* to be those ≤ 30 mm in radius. For each site where stars were measured in LTM plots, the total number of juveniles counted per survey during annual sampling, or the mean total number for semiannual surveys, was compared over the period of study.

Estimating survivorship of juvenile *P*. *ochraceus* in the field is challenging, largely because small sea stars (≤20 mm radius) are cryptic and difficult to count accurately; however, as stars grow larger, they become much more obvious to observers. If we assume our ability to detect the smallest size classes of stars did not change over time (i.e., we always failed to detect the same fraction of the population ≤20 mm in radius), we can estimate the additive impact of SSWD on juvenile survivorship by comparing transition rates of size classes during periods pre- and post-onset of SSWD. Field growth rates for *P*. *ochraceus* are difficult to estimate because the stars are exceedingly challenging to tag. Sewell and Watson [35] followed a distinct pulse of *P*. *ochraceus* recruits over a 2 year period, and estimated that individuals approximately 20 mm in radius (est. age = 9 months) grew to approximately 60 mm in size after 1 year. We used this published estimate of growth rate in our calculations, but among-site differences in rate of growth are certainly possible due to variation in factors such as prey availability and seawater temperature. However, our primary goal was not to calculate growth rates, but rather assess whether SSWD affects the transition rate from one size class (10–20 mm) to another (50–60 mm) (i.e. survivorship). We used these values to determine whether transition rates from our smallest size classes (10–20 mm) to the size class after an estimated 1 year of growth (50–60 mm) was impacted by SSWD. Juvenile mortality due to SSWD was estimated as follows:

*N*_*1*,*i*, *j*_ = *# P*. *ochraceus 10–20 mm (stage 1 “recruits”) at time i and at site j*
*N*_*2*, *i+1*, *j*_ = *# P*. *ochraceus* *50–60* *mm (stage 2 “recruits” after 1 year of growth) at time i + 1 year at site j*

In the period prior to onset of SSWD (pre), *N*_*2*,*i+1*, *j*_ *= N*_*1ij*_*D*_*j*_*S*_o,j_ where *D*_*j*_ *= relative difference in detectability between stage 1 and stage 2 recruits at site j*, *and S*_*o*,*j*_ *= ordinary survivorship between stage 1 and stage 2 for site j*. We assume that *D* and *S*_*o*_ are constants. This means that for year = *i*:

$$\frac{N_{2,i+1,j}}{N_{1,i,j}}=D_{j}S_{o,j}$$ (1)

During the period following onset of SSWD in populations of *P*. *ochraceus* (post), *N*_*2*,*i+1*, *j*_ *= N*_*1ij*_*D*_*j*_*S*_*o*,*j*_*S*_*w*,*j*_, where *S*_*w*,*j*_ *= change in survivorship due to wasting between stage 1 and stage 2 at site j*.

$$\frac{N_{2,i+1,j}}{N_{1,i,j}}=D_{j}S_{o,j}S_{w,j}$$ (2)

Therefore for any site and pre/post year combination:

N2,i+1,j,postN1,i,j,postN2,i+1,j,preN1,i,j,pre=DjSo,jSw,jDjSo,j=Sw,j (3)

And juvenile mortality due to wasting is:

$$M_{w,j}=1-S_{w,j}$$ (4)

We can calculate all the terms on the left side of Eq 3 (the N terms) for all sites where there were years with recruits in both the *pre* and *post* periods, which means we can get estimates of S_W_ and therefore also M_W_ (total number of informative sites = 35). Because the values were extremely non-normal, we bootstrapped values 2500 times to generate a distribution of possible means, which allowed estimation of the overall median and confidence intervals.

## Results

### Geographic patterns of decline and potential role of density

**Fig 2:**
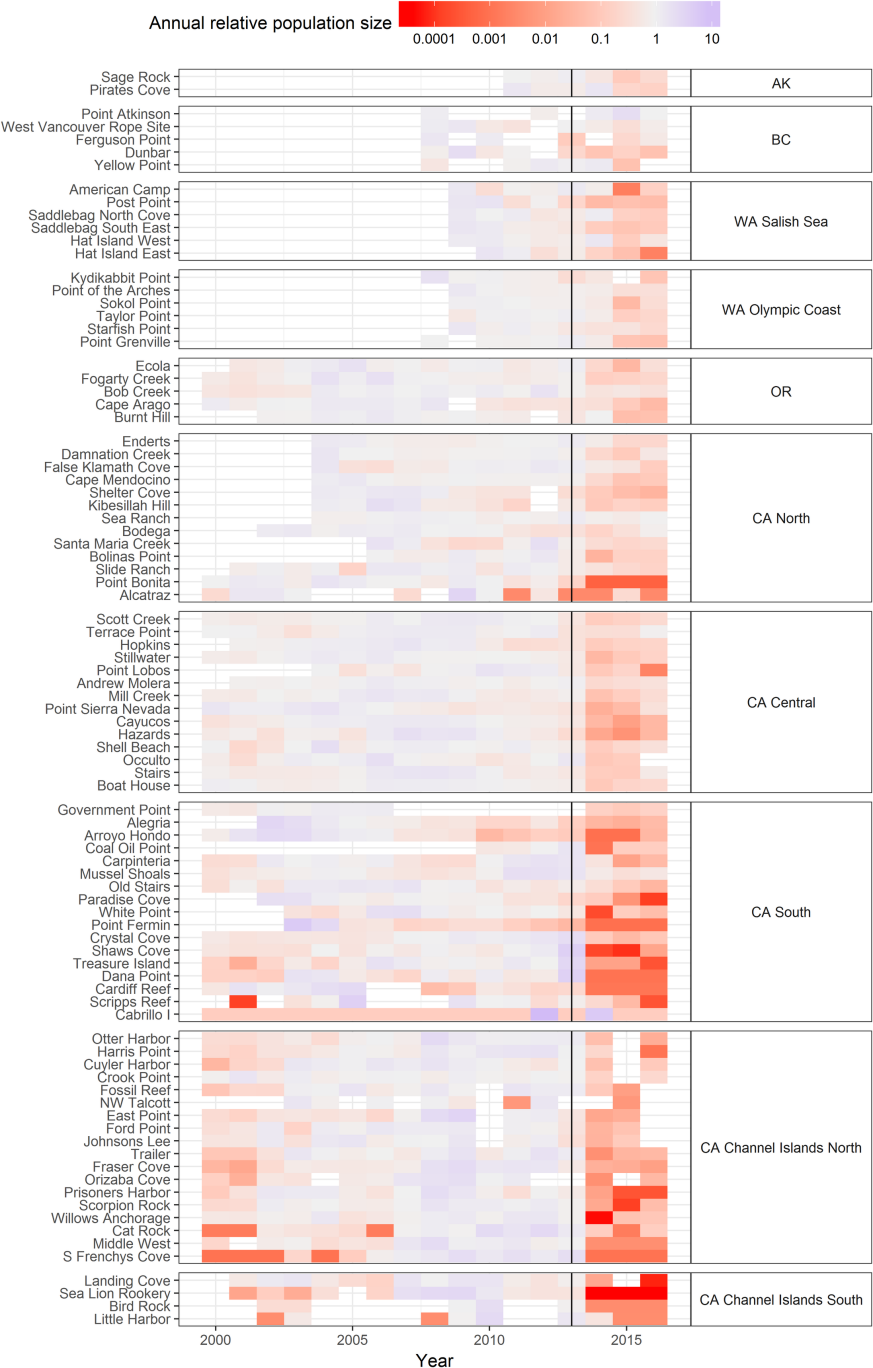
Heat map showing annual changes in abundance of *P*. *ochraceus* for each site relative to the long-term mean. The annual relative population size was calculated by dividing the total number of adult *P*. *ochraceus* (>30 mm radius) counted within long-term permanent plots for a given year (or mean count in years with >1 survey year^-1^) by the long-term annual mean number of stars counted at a given site through 2013. 2013 is indicated by a vertical line, and separates pre-SSWD years from post-SSWD years. Unshaded cells represent years when surveys were not done.

Following the onset of SSWD in 2013, substantial declines in intertidal *P*. *ochraceus* abundance occurred at nearly all sites, from Alaska to southern California (Fig 2). Regional declines had been observed prior to 2013, particularly at sites in southern California and the Channel Islands (e.g., 2000–2001) where populations were reduced by earlier disease events (see [24,36]), but the broad geographic coherence of declines in 2014–2015 represent disease impacts unprecedented in geographic scale. While declines were ubiquitous post-onset of SSWD, the timing and severity of the declines showed some smaller-scale regional variation. It is important to note that the patterns described here are based on population declines documented by our annual or semiannual surveys, not on when disease symptoms were first observed. The frequency of our monitoring was designed to document long-term population trends and is too crude to assess patterns of disease emergence. Populations at all California sites were in decline by 2014, with declines beginning at some sites in 2013. Declines generally occurred earlier and were typically more severe in the southern regions (southern California and the Channel Islands) as compared to regions further north. Percent decline of stars in adult size classes (>30 mm) exceeded 75% at all but one southern site, and was ≥ 99% at over half of the 39 sites in the southern regions. By contrast, declines generally occurred later and were less extreme in northern regions (San Francisco, CA north to Alaska), although timing of decline varied substantially among northern regions, and even among sites within these regions. In the north, population crashes tended to be less severe than in regions further south. *P*. *ochraceus* populations declined by at least 75% (compared to pre-SSWD means) at many (80%) sites, but only 2 of the 36 sites exhibited a decline > 99%. Among the northern regions, crashes were most severe at sites within San Francisco Bay and also the Salish Sea region of Washington.

There was no relationship between severity of decline and the interaction between density and region suggesting that any relationship between density and severity was unaffected by region (F_1, 50_ = 0.4544, p = 0.8818). Hence we dropped the interaction term from the model and ran a reduced model containing only the main effects of region and density. In the reduced model there was no effect of density (F_1, 58_ = 0.0063, p = 0.9372), and a significant effect of region (F_1, 58_ = 1.49, df = 1,58, p = 0.043). There was no evidence that higher sea star densities were associated with more dramatic declines either within or among regions. Within the northern California region, for example, Damnation Creek, False Klamath Cove, and Bodega were among the most densely populated sites, but *P*. *ochraceus* decline at the less densely populated Alcatraz site was much more severe (Fig 2). Among regions, the generally densely populated north experienced only modest declines relative to the less densely populated south.

### Sea surface temperature

**Fig 3:**
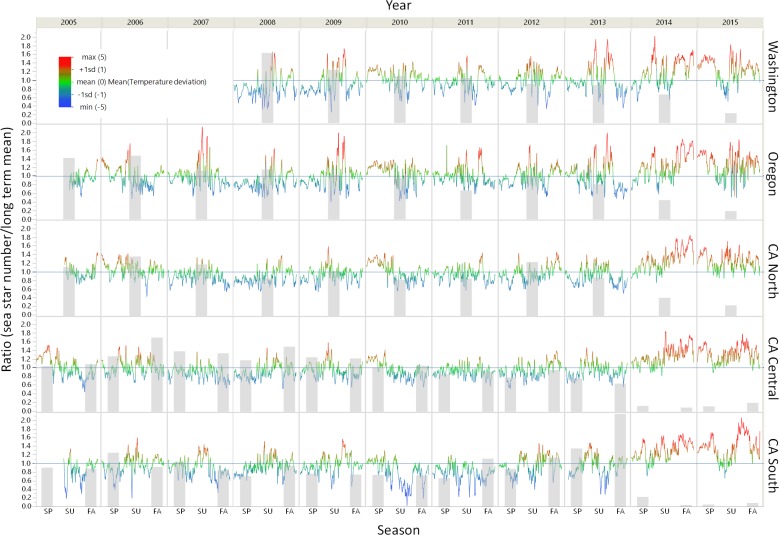
Deviations of daily sea water temperature from long-term means (lines) overlaid on annual (WA, OR, CA North) or semiannual (CA Central, CA South) *P*. *ochraceus* abundance (bars) relative to long-term mean. Temperature deviations were displayed using a weekly smoother to emphasize longer-term patterns (rather than daily fluctuations). Sea star abundance ratios are averaged across all sites within each region for a given season (SP = spring [Feb-Apr], SU = summer [May-Aug], FA = fall [Sep-Nov]). Horizontal lines where ratio = 1 were included to illustrate deviations from long-term mean.

Sustained periods of anomalously high seawater temperatures were documented in 2014 and 2015 across all regions, and declines in populations of *P. ochraceus* were associated with these periods of elevated temperatures at some, but not all, regions (Fig 3). The relationship between temperature and sea star decline was not formally tested because the temporal scale of our population surveys was too coarse to capture the temporal scale at which *P. ochraceus* responds to its environment (e.g., [33]). On the Washington outer coast, symptomatic stars were first noted in June 2013, following a period of anomalously high seawater temperatures, and symptomatic stars were found frequently during periods of elevated temperatures in 2014–2015 [37]. In Oregon, SSWD was first documented in April 2014 [14] between brief periods of elevated seawater temperatures in March (just prior to SSWD emergence) and May (prior to our summer survey when symptomatic stars were observed). However, temperatures in Oregon were not consistently elevated (relative to long-term means) until late 2014 / early 2015 (Fig 3). Because *P. ochraceus* surveys in northern California are primarily conducted in the summer, the precise timing of initial SSWD emergence in this region is unknown, but population declines had occurred by summer 2014, when symptomatic stars were observed. Seawater temperatures in northern California in spring and early summer of 2014 were slightly elevated above the long-term mean, but less so than in some previous years (e.g. 2010, Fig 3). Finally, diseased stars were first noted in central and southern California in fall 2013, long before increased seawater temperatures were recorded, and population declines attributed to SSWD began prior to the onset of elevated seawater temperatures. While there is no strong evidence from our data to support elevated seawater temperatures as a factor contributing to the initial emergence of SSWD, sustained, anomalously high seawater temperatures in 2014 and 2015 might have exacerbated the disease’s impact, as sea star declines continued in all regions.

### Juvenile abundance and impact of SSWD on juvenile survivorship

**Fig 4:**
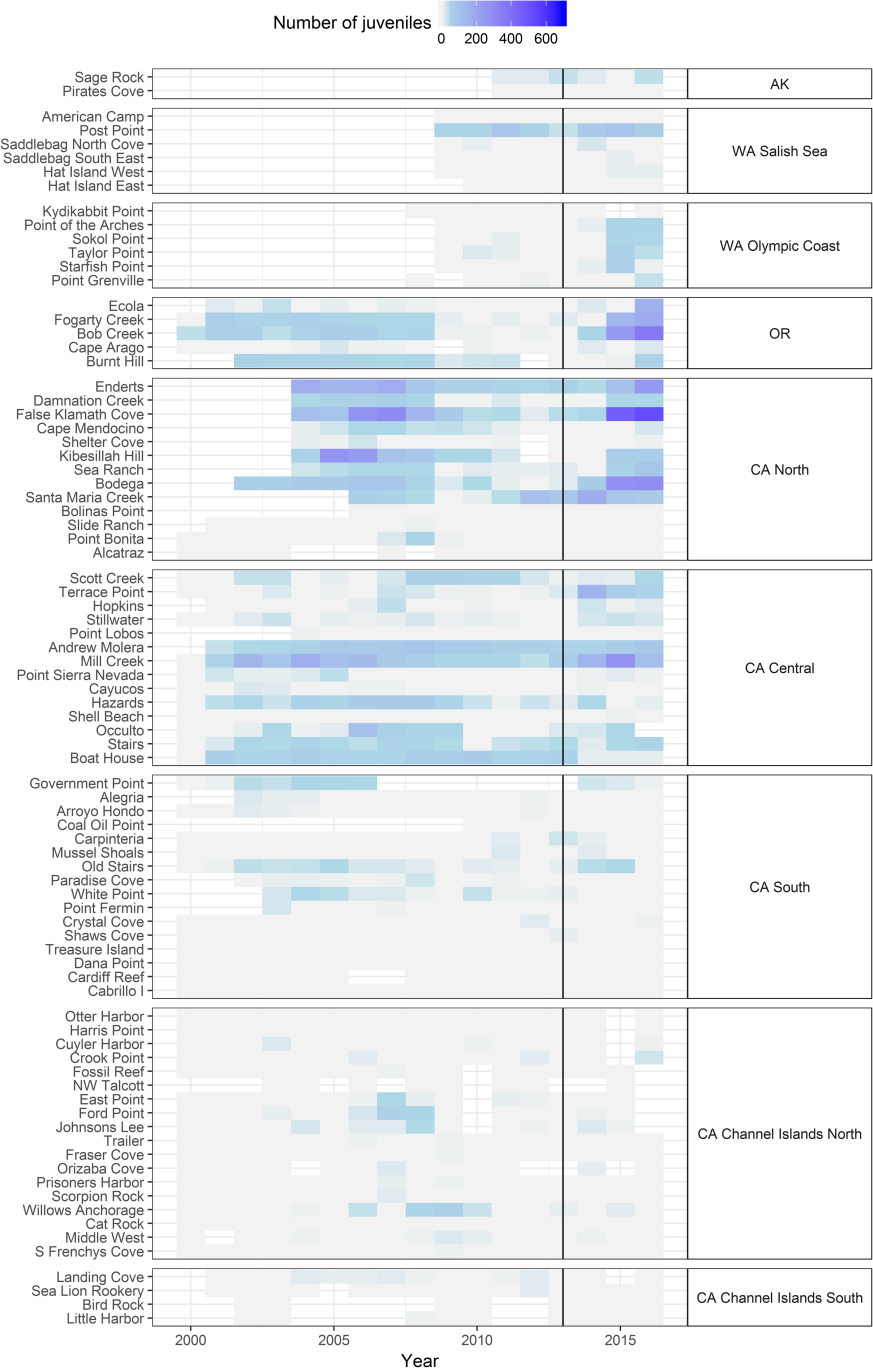
Total number of juvenile *P*. *ochraceus* (“radius” ≤ 30 mm) counted within long-term permanent plots at each site over time. For sites that were sampled > 1 time year^-1^, the mean total number for that year is displayed. 2013 is indicated by a vertical line, and separates pre-SSWD years from post-SSWD years. Unshaded cells represent years when surveys were not done.

Our LTM data suggest there may be two very different recruitment regimes north and south of the biogeographic break at Point Conception. Juvenile (≤ 30 mm radius) *P*. *ochraceus* abundance exhibited a strong spatial signal, with high numbers of juveniles recorded at sites ranging from Washington to central California, and few to no juveniles at sites on the southern California mainland and the California Channel Islands (Fig 4). This pattern is evident in both the pre- and post-onset of SSWD periods, but was particularly strong in the post-onset years. Juveniles were also rare in Alaska, but with only two sites, it is difficult to draw conclusions about the region as a whole. In regions north of Point Conception, several sites appeared to be consistently favorable for recruitment in the period pre-onset of SSWD, with relatively high numbers of juveniles recorded across successive years (e.g. Post Point, Fogarty Creek, Enderts, Mill Creek, Fig 4). During the period post-onset of SSWD, sites where juveniles were consistently present prior to the disease event tended to have the largest numbers of juveniles, but juveniles were also recorded at sites where they had previously been rare or absent (e.g., Washington Olympic Coast). Thus, while consistent historic recruitment tended to be a good predictor of high post-SSWD recruitment, it was not a prerequisite. All-time high counts of juveniles were recorded in LTM plots in the post-SSWD period (2014–2016) at 16 of 46 sites in the regions north of Point Conception as opposed to 2 of 40 sites in regions to the south. In addition, these all-time high, post-SSWD juvenile counts at northern sites tended to be orders of magnitude greater (63% had counts > 100, max count = 702 juveniles) than at southern sites (counts = 6 and 20 juveniles).

Low rates of transition between juvenile size classes in the post-onset SSWD period suggest that the disease might have negatively impacted juvenile survivorship. The median estimated juvenile mortality rate due to SSWD (on top of the ordinary, pre-SSWD mortality rate) across all informative sites was 88.5% (2-tailed 95% CI = 65.4%-93.5%). This means that compared to the pre-SSWD period, approximately 90% more juvenile stars on average died before reaching the next year’s size class when SSWD was present in the population.

## Discussion

Prior to 2013, regional disease outbreaks had been implicated in punctuated and spatially isolated declines in *P*. *ochraceus* abundance along the North American Pacific coast from Baja California, Mexico [24] to British Columbia, Canada [23]. However, unlike these previous regional events, in 2014–2015 we documented synchronous declines in *P*. *ochraceus* populations across multiple biogeographic regions—a marine disease outbreak of unprecedented geographic scale and magnitude. *P*. *ochraceus* individuals do move between the intertidal and subtidal zones, and our surveys did not extend into the subtidal zone; thus it is possible that our data overestimated population declines at our sites if *P*. *ochraceus* individuals persisted in large numbers in subtidal refugia. However, two lines of evidence suggest that this is unlikely. First, the severe declines that have been documented for asteroids (including *P*. *ochraceus*) in subtidal habitats within the range of the SSWD outbreak give no indication of a subtidal refuge from the disease (e.g., [15,38]). We have made similar direct observations of declines in numbers of subtidal asteroids at a subset of sites where both intertidal and subtidal surveys were conducted (P.T. Raimondi, M.H. Carr, B.G. Miner, and Channel Islands NP unpublished data). Second, the persistently low abundance of individuals observed in our post-onset of SSWD intertidal surveys through 2016 lends no support for the conclusion that our sites harbored pools of healthy subtidal *P*. *ochraceus* individuals and thus that our intertidal surveys over-estimated the degree of disease impact at our sites.

Over the past several years, apparent increases in the frequency of marine disease outbreaks [4] have led to repeated calls for a ‘multi-step’ approach to disease ecology (e.g., [2,25–27]). Across the board, these calls highlight the importance of surveillance of potentially susceptible populations to enable quick detection of disease outbreaks, and subsequently emphasize that the ultimate goal is to predict outbreaks before they appear or spread. In hindsight, our data suggest that several aspects of the SSWD event defied prediction. Although “wasting disease” had been documented in *P*. *ochraceus* before 2013, the etiology of those outbreaks is not known [23,24,36] and may well have varied among locations and outbreak events [23]. Similarly, our lack of understanding of the agent or agents responsible for the current SSWD outbreak hindered our ability to make predictions about the timing and pattern of the outbreak. In the absence of an identified pathogen, outbreak predictions must be based on more generalized patterns or models of disease transmission. For example, one might predict that pattern of decline could be explained by density-dependent disease dynamics. Our geographically extensive data set shows that the intensity of the impact of SSWD was not uniform across the entire affected area, with proportionally greater population declines in the lower density southern regions (mainland southern California and the California Channel Islands) than in the higher density regions of northern California, Oregon, and Washington. The degree of population decline was unrelated to pre-outbreak *P*. *ochraceus* density and demonstrated substantial site-to-site variation within regions. This lack of association between impact and density (and therefore of a potential tool for predicting impact) contrasts with the patterns detected in other well-documented disease events in which the degree of impact was directly correlated with population density [1,4]. The recent suggestion of a linkage between susceptibility to SSWD and genotype within a documented polymorphism [39] might indicate that SSWD transmission is better modeled as a frequency-dependent process, in which transmission rates depend on the relative frequency of susceptible individuals in the population rather than on population density *per se* [29]: this is an intriguing avenue for continued investigation.

Heavy emphasis has been placed on the potential for scientists to use temperature data to develop tools to forecast marine disease outbreaks and predict impact to natural populations [27], as high seawater temperatures are widely associated with high prevalence and severity of marine diseases [2,8,27]. However, our data indicate that the role of temperature in the initiation or intensification of the SSWD outbreak is still unclear. Characterizing the effects of temperature on disease outbreaks for organisms in the intertidal zone is complicated by the change in medium that accompanies changing tides. During emersion at low tide, the body temperature of a sea star is determined by multiple factors including air temperature, shading, angle of incidence to sun, rock type, humidity, wind speed, body shape, size, and behavior [40–42]. Thus, body temperatures cannot be accurately characterized by proxies such as air or substratum temperature, but this instead requires specialized thermal mimics [41]. Because we do not have these data, it is difficult to know if body temperatures at low tide played a role in the SSWD outbreak. By contrast, seawater temperature is a relatively easy metric to measure with existing temperature logger technology, and provides an accurate proxy of *P*. *ochraceus* body temperature while submerged (at high tide). During submersion, small objects and organisms rapidly equilibrate to the temperature of the surrounding ocean water. Water temperature strongly influences the metabolic rate of *P*. *ochraceus* [43], and plays an important role in thermoregulation during low tide [42]. Some lab studies have shown that short-term exposure to warmer water elevates SSWD prevalence, increasing the proportion of symptomatic individuals and the severity of the outbreak [23], while other studies indicate that temperature affects the onset of symptoms for *P*. *ochraceus* juveniles, but not for adults [13]. Cooler water temperatures can slow progression of the disease, but not prevent mortality [13,20]. Two regional studies that analyzed *P*. *ochraceus* population data at finer geographic and temporal scales than our study reached different conclusions about the role of temperature in this epidemic. Eisenlord et al. [13] reported that anomalous (elevated) seawater temperatures were linked to increased probability of SSWD presence at their sites on the San Juan Islands, WA. By contrast, Menge et al. [14] recorded cooler than normal mean monthly seawater temperatures in the period preceding their first observations of stars showing signs of SSWD in Oregon. The relationship presented here between seawater temperature anomalies and abundance of *P*. *ochraceus* relative to the long-term mean suggests that on a large geographic scale, anomalously high temperatures are unlikely to have played a role in disease onset, as in most regions, symptoms appeared in populations prior to periods of elevated water temperature. However, our data do show that the intensity of the outbreak was elevated in warmer-water southern regions relative to cooler northern areas. While our data add to the body of literature which indicates that SSWD intensity might be affected by water temperature, they reinforce the conclusion by Maynard et al. [27] that the relationships between water temperature and SSWD onset and impact are complex and still largely undetermined.

Until we have an understanding of the etiology and mode of transmission of SSWD, our ability to effectively model future outbreaks is critically limited [29]. This highlights the importance of surveillance as one facet of an integrated approach to disease ecology. This SSWD epidemic is a perfect example of an ‘ecological surprise’ [44] that underscores the need for long-term ecological and environmental studies (LTEES). Multi-year monitoring programs allow researchers to characterize the range of natural variation in populations and thus to distinguish anomalies from natural fluctuations [45–46]. In addition, the pattern of sustained regular observations puts researchers in a position where they can witness rare events that may otherwise go unnoticed with a snapshot or short-term study [44]. This context and regularity provide a critical link between ‘basic’ and ‘applied’ science when an epidemic occurs, as effective disease management starts with ‘routine tasks’ that lead to early disease detection and communication of results [8]. Diseased sea stars were first reported by researchers at Olympic National Park, Washington, who had been collecting population data on *P. ochraceus* for several years as part of the MARINe network. Established avenues for communication among network partners allowed the rapid addition of specific disease surveillance activities to their ongoing coordinated monitoring efforts across a wide geographical range. Furthermore, communication beyond the MARINe network facilitated exactly the sort of rapid and effective outreach to the public, the media, and additional researchers that Groner et al. [8] recommend as the model response to disease outbreaks. For example, the addition of open access disease reporting and mapping tools through a dedicated web address (www.seastarwasting.org) linked to the MARINe website and the development of citizen science disease detection materials allowed free exchange of information among scientists, managers, policy-makers, and the public. Yet despite the documented value of LTEES both for the field of ecology [44] and as the first critical step in responding to disease emergencies [8], funding to maintain existing LTEES is dwindling, and few new LTEES are being established [44]. Furthermore, even relatively well-supported LTEES such as MARINe do not have access to emergency funding to rapidly respond to ecological disasters. Such funding would have allowed for more frequent sampling after SSWD was first detected, which would have provided finer temporal scale information about disease emergence and sea star decline that could be more closely paired with environmental factors such as temperature. Without the collection of coordinated environmental and biological data, we will be unable to develop large-scale forecasting tools that can allow researchers and managers to rapidly respond to future marine disease emergencies and help shape policies that could prevent or lessen the impact of these events [27].

Another essential function of LTEES, particularly those occurring at a broad geographic scale such as this sea star monitoring effort, lies in providing the data necessary for predicting population recovery rates after an impact has occurred. Recruitment and survivorship to reproductive size are important components of population recovery estimates, but are often not well documented for species that are not commercially harvested. The data presented here demonstrate that recruitment and survivorship for *P*. *ochraceus* varies geographically, at both broad and localized scales, and also highlight the challenges of collecting basic life history data in the dynamic intertidal zone, where being cryptic is essential to juvenile survivorship, and unhealthy individuals do not persist for long. Because we defined recruits as *P*. *ochraceus* ≤ 30 mm in radius, stars in even our smallest size classes are likely several months old. Therefore, we cannot determine whether differences in juvenile abundance at either broad or localized scales resulted from differential settlement, differential post-settlement mortality, or both. Sewell and Watson [35] reported extremely low survivorship of *P*. *ochraceus* recruits smaller than 40 mm, exceeding 97% mortality over one year in a population with no reported symptoms of disease. Laboratory studies have suggested that the progression of SSWD occurs more rapidly in juvenile *P*. *ochraceus* than in adults [13]; thus during and after the SSWD outbreak, newly settled recruits might not have survived to a size at which they were detectable in our semiannual surveys. Our results suggest that juvenile survivorship was substantially reduced by the presence of disease when pre- and post-onset of SSWD periods were compared. If SSWD was more virulent in the southern regions, this differential mortality across the Pt. Conception boundary could explain the difference in recruitment in the north and the south. Unfortunately, juvenile abundances were too low at southern sites to test for a difference in juvenile mortality between northern and southern regions post-SSWD onset and to our knowledge no data are available to allow comparisons of virulence across regions. Regardless of the mechanism (differential settlement, differential mortality, or both), our data show a clear difference in recruitment between ‘north’ and ‘south’ on this larger geographic scale, yet even within the ‘high recruitment’ northern regions, patterns of recruitment were extremely variable among sites, with no distinct latitudinal pattern.

Our geographically broad data set provides important context for recruitment patterns previously highlighted by more regional studies. Along the Oregon coast, Menge at al. [14] documented ‘unprecedented’ numbers of juvenile recruits at seven long-term monitoring sites in 2014–2015. Our data from Fogarty Creek, OR (one of the sites sampled by Menge et al. [14]) also show high numbers of juveniles during this period. However, of our remaining four Oregon study sites, only one had higher than normal recruitment after the SSWD outbreak, resulting in a total of eight out of eleven sites in Oregon with higher than typical levels of ‘post-outbreak’ recruitment. A similar ‘spatially mixed’ pattern of recruitment within a region was reported by Eisenlord et al. [13] in Washington. Overall, because of this contrast between the large-scale geographic break (north/south) and smaller scale variability, the data suggest that a “general” pattern of recruitment for *P*. *ochraceus* may be difficult to describe, and consequently that our ability to predict the trajectory of recovery for any given site or region is limited. Yet despite patchy recruitment patterns and high mortality rates for juveniles, some fraction of the *P*. *ochraceus* that recruited after the SSWD outbreak did survive and transition into larger size classes (S2 Fig). The ‘spatially mixed’ distribution of low and high recruitment sites in northern regions could be viewed as encouraging, because if populations at high recruitment sites do recover, they could potentially serve as sources of larvae for neighboring sites [35]. Harley et al. [47] found little evidence of genetic differentiation among *P*. *ochraceus* populations from Alaska to southern California, indicating there is no barrier to genetic connectivity. However, the results of oceanographic modeling indicate dispersal events are generally more restricted and driven largely by surface currents, which is more relevant on an ecological scale (e.g., [48]). We echo the statements of other authors in that the evidence of recruitment and persistence of juvenile *P*. *ochraceus* at some sites leaves us ‘hopeful for recovery’ [13] in at least a portion of the sea star’s range. However, if these larger geographic recruitment trends continue, the outlook for recovery of *P*. *ochraceus* populations in the southern regions is poor, as numbers of juveniles recorded at the southern sites are orders of magnitude smaller than those recorded at many northern sites.

Will the absence or sustained anomalously low abundance of this important intertidal predator ripple through the food web and result in long-term impacts at southern sites? *P*. *ochraceus* is well-known as the original keystone species [7], but its’ ability to play a keystone role in rocky intertidal systems hinges on the community role of the foundation species *Mytilus californianus*, which in turn depends on environmental factors such as the degree of wave action and sand influence at a site [19]. For example, mainland southern sites in Orange County are heavily sand influenced with low wave forces [49] and patchy, monolayered *M*. *californianus* beds (authors’ unpublished data: www.pacificrockyintertidal.org), a set of conditions under which *P*. *ochraceus* would not be expected to exert keystone predator effects [19]. It remains to be seen how the severe and persistent declines of *P*. *ochraceus* will impact the structure and make-up of rocky intertidal communities at this never-before documented scale. We have clues from small-scale studies [7,50], but the vast amount of coastline impacted by the current wasting event encompasses much more variation in the factors contributing to community structure than could ever be tested experimentally, and it is likely that our understanding of the role this keystone predator plays in shaping community structure will be revised and improved in the coming years.

## Supporting information

S1 Fig **Regional maps of study sites:** Panel A in S1 Fig) Alaska sites, Panel B in S1 Fig) British Columbia sites, Panel C in S1 Fig) Washington sites, Panel D in S1 Fig) Oregon sites, Panel E in S1 Fig) Northern California sites, Panel F in S1 Fig) Central California sites, Panel G in S1 Fig) Southern California mainland sites, Panel H in S1 Fig) Southern California Channel Island Sites.
(PDF)

S2 Fig Size frequency graph displaying changes in size distribution of *P*. *ochraceus* within permanent plots at one representative site (Terrace Point, site #39). Sizes are radial measurements (see methods) and surveys are labeled as spring (Feb-Apr) or fall (Oct-Nov) samples for a given year. Bubble size represents number of individuals recorded for each size bin. Size frequency graphs can be found for all sites here: http://www.eeb.ucsc.edu/pacificrockyintertidal/sites/sites-target-species.html#pisaster
(PNG)

S1 Table Names and locations of all sea star sites. Site numbers refer to the maps in S1A–S1H Fig. Monitoring groups include: Sitka Sound Science Center (SSSC), University of California Santa Cruz (UCSC), University of British Columbia (UBC), Olympic National Park (ONP), Padilla Bay National Estuarine Research Reserve (PBNERR), Olympic Coast National Marine Sanctuary (OCNMS), Redwoods National and State Park (RNSP), Point Reyes National Seashore (PORE), Golden Gate National Recreation Area (GOGA), University of California Los Angeles (UCLA), California State University Fullerton (CSUF), California State Polytechnic University Pomona (CPP), Cabrillo National Monument (CABR), Channel Islands National Park (CHIS).
(PDF)
