# Supplementary figures and images for: Large-scale impacts of sea star wasting disease (SSWD) on intertidal sea stars and implications for recovery
Source: PLoS One. 2018 Mar 20;13(3):e0192870. doi: 10.1371/journal.pone.0192870 (PMC5860697; doi:10.1371/journal.pone.0192870)

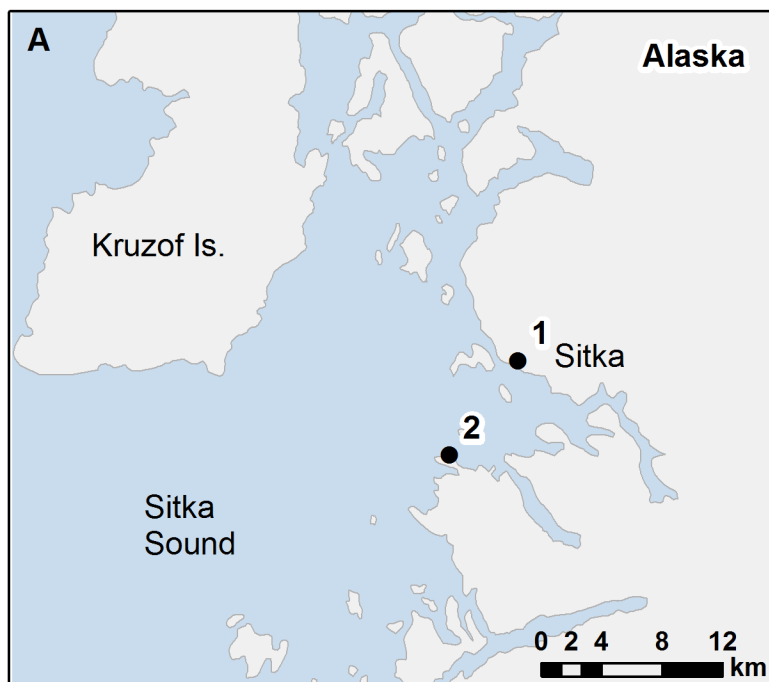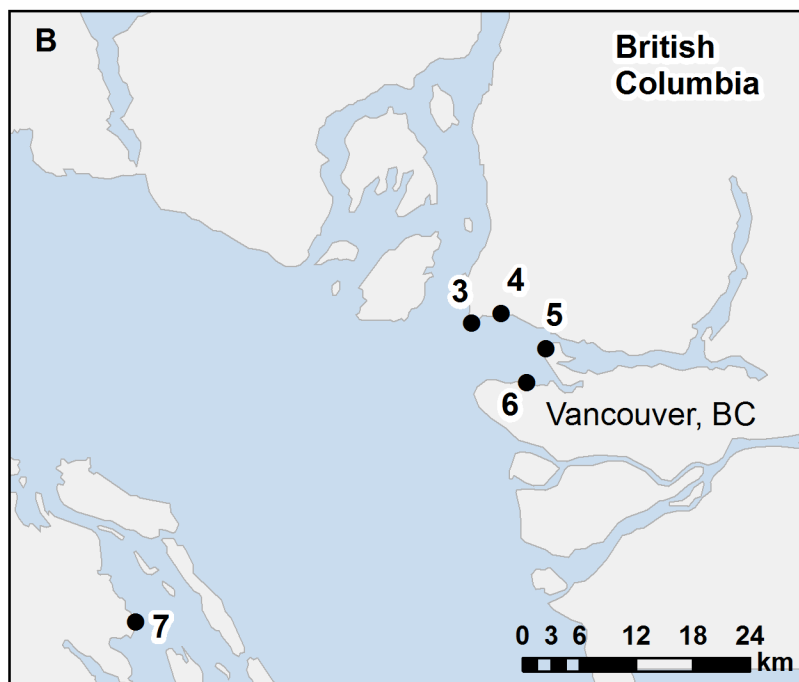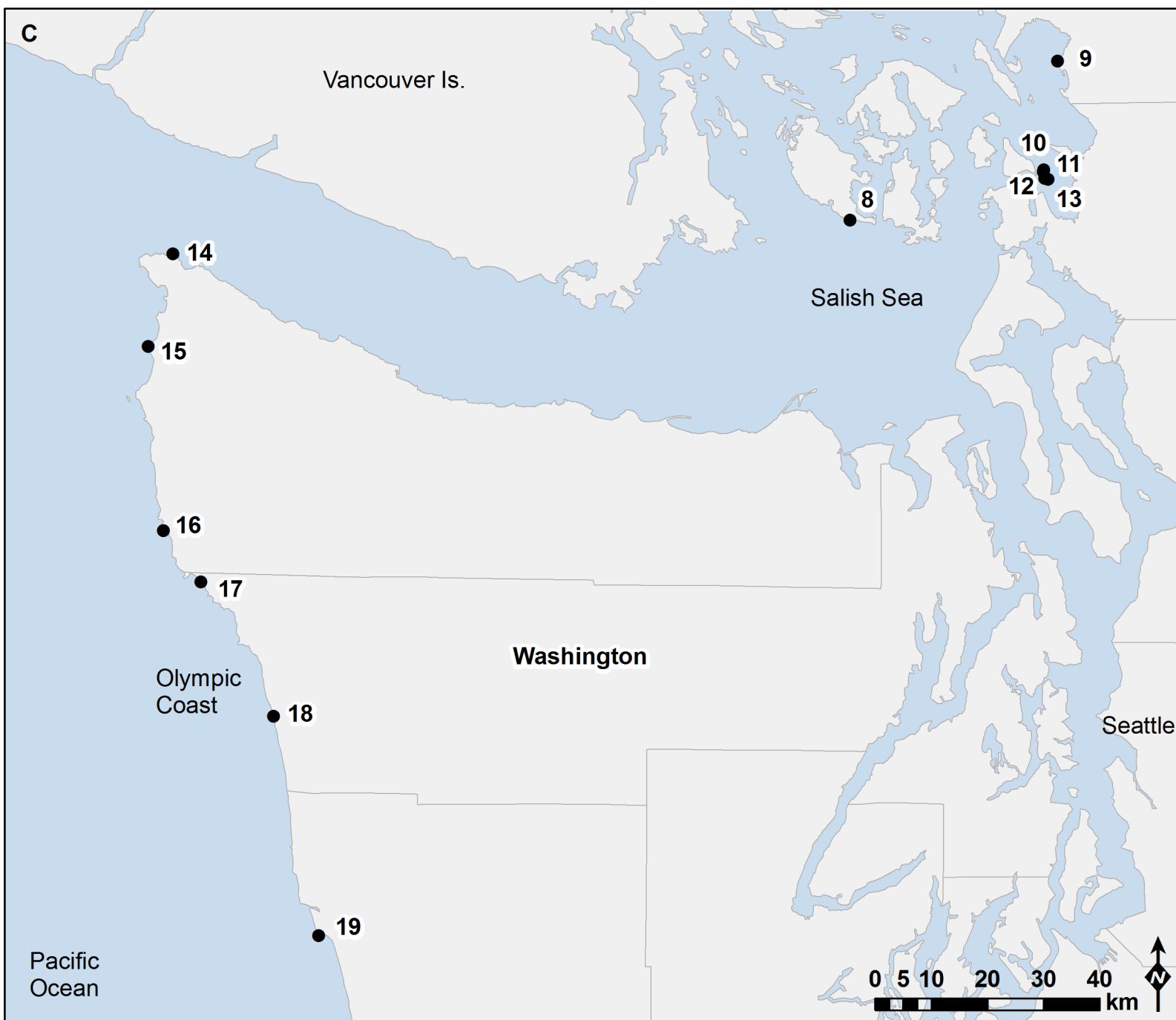

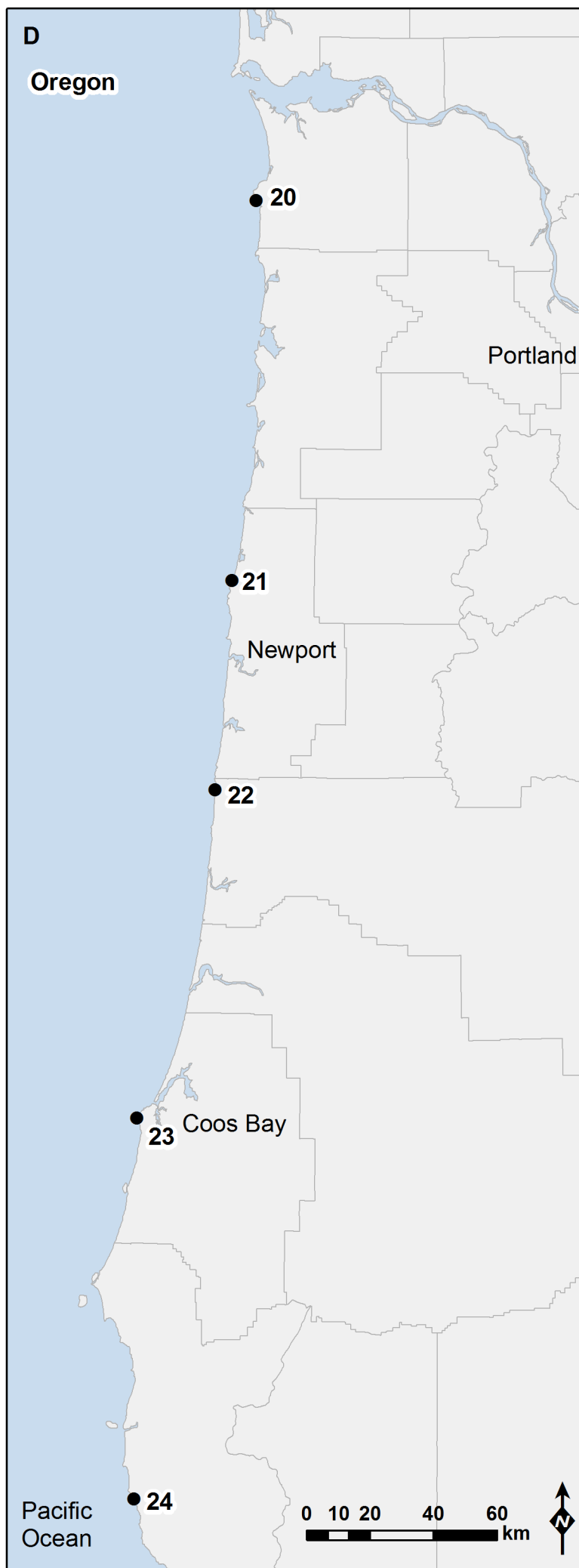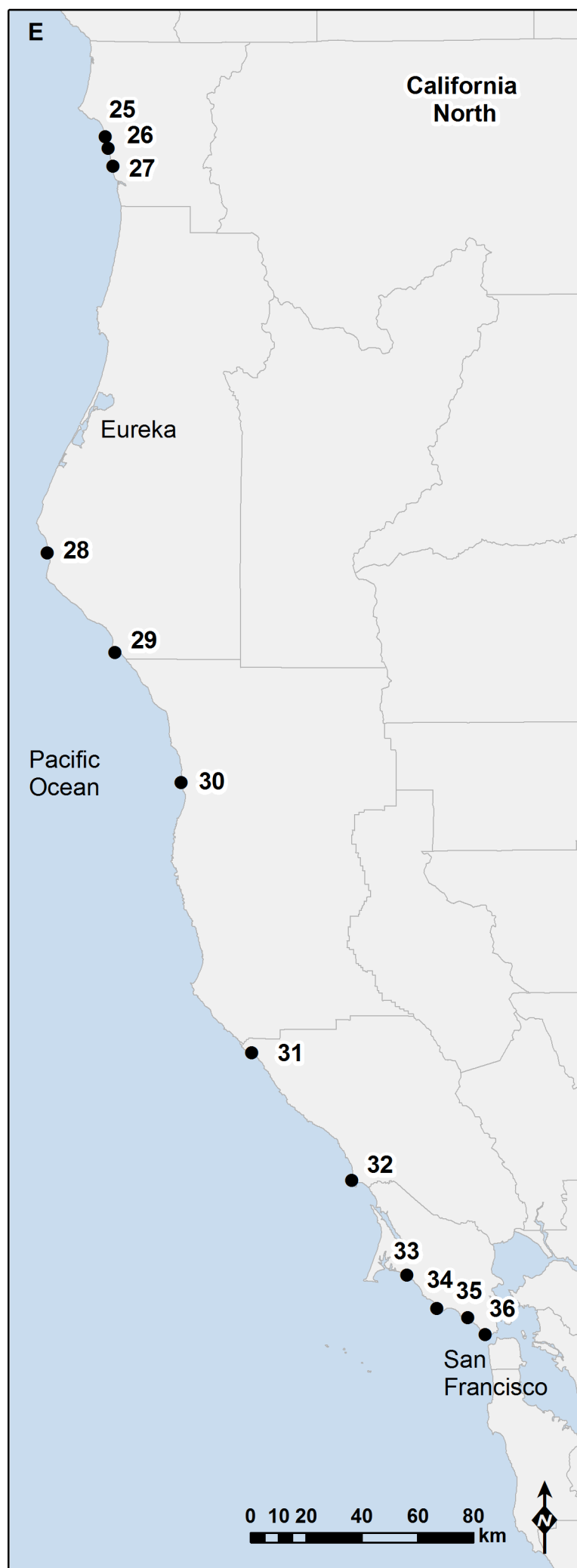

F

California  
Central

37

San  
Francisco  
Bay

38

39

Monterey  
Bay

40

41

42

43

44

45

46

47

48

49

50

51

Santa Barbara

Pacific Ocean

0 10 20 40 60 80 km

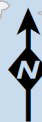

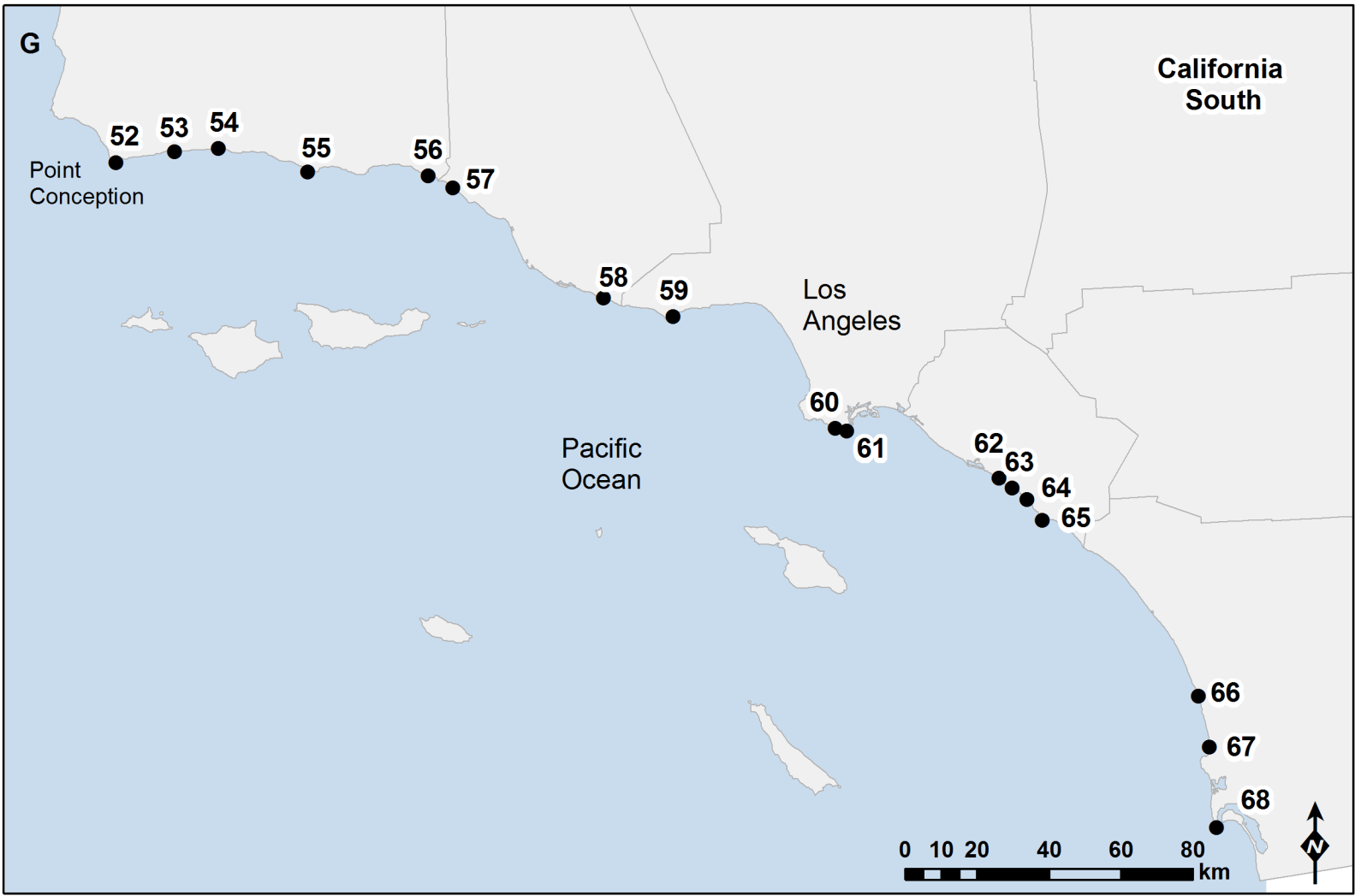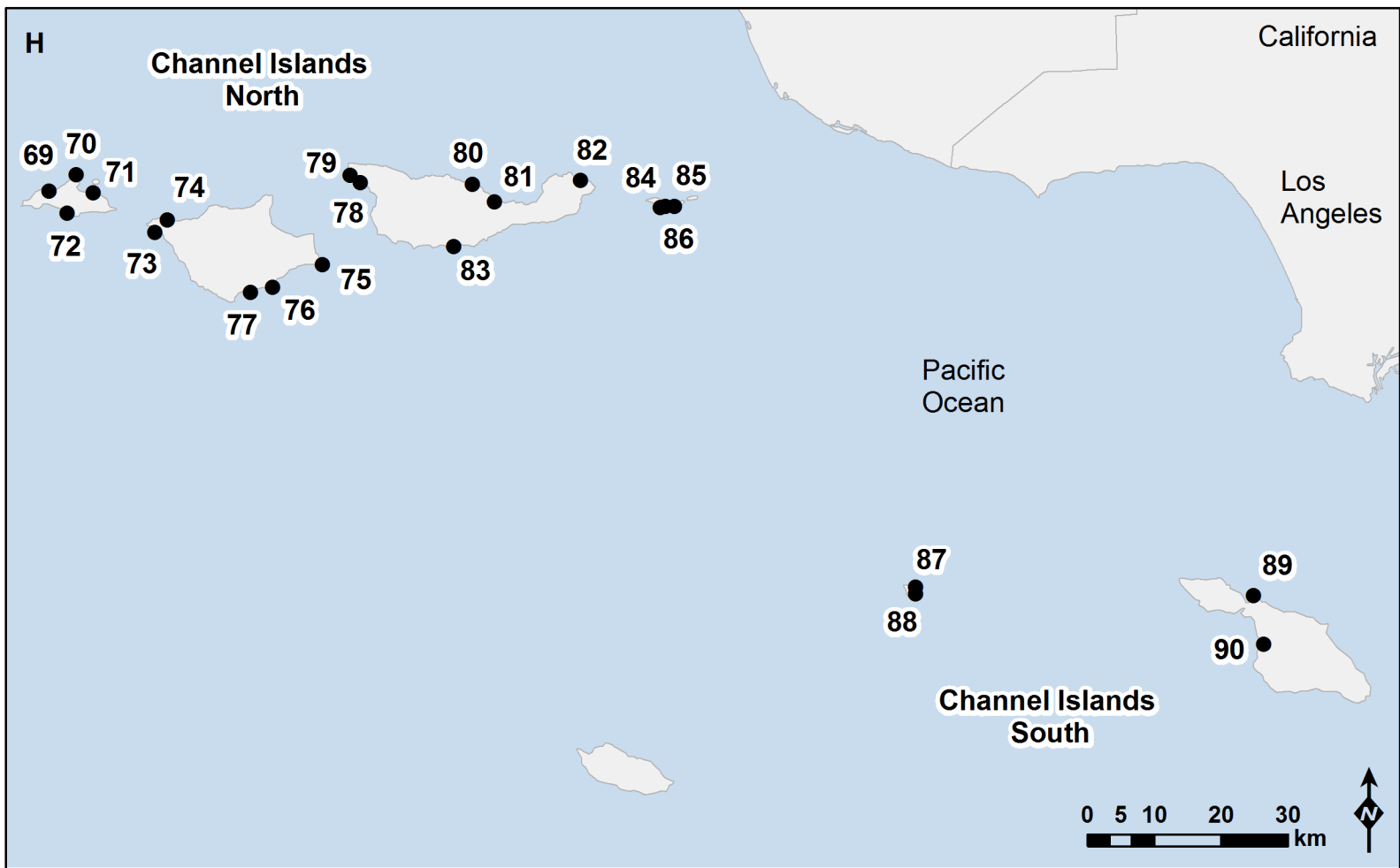

Supplement: S1 Fig — Regional maps of study sites: Panel A in S1 Fig) Alaska sites, Panel B in S1 Fig) British Columbia sites, Panel C in S1 Fig) Washington sites, Panel D in S1 Fig) Oregon sites, Panel E in S1 Fig) Northern California sites, Panel F in S1 Fig) Central California sites, Panel G in S1 Fig) Southern California mainland sites, Panel H in S1 Fig) Southern California Channel Island Sites. (PDF) [file pone.0192870.s001.pdf]

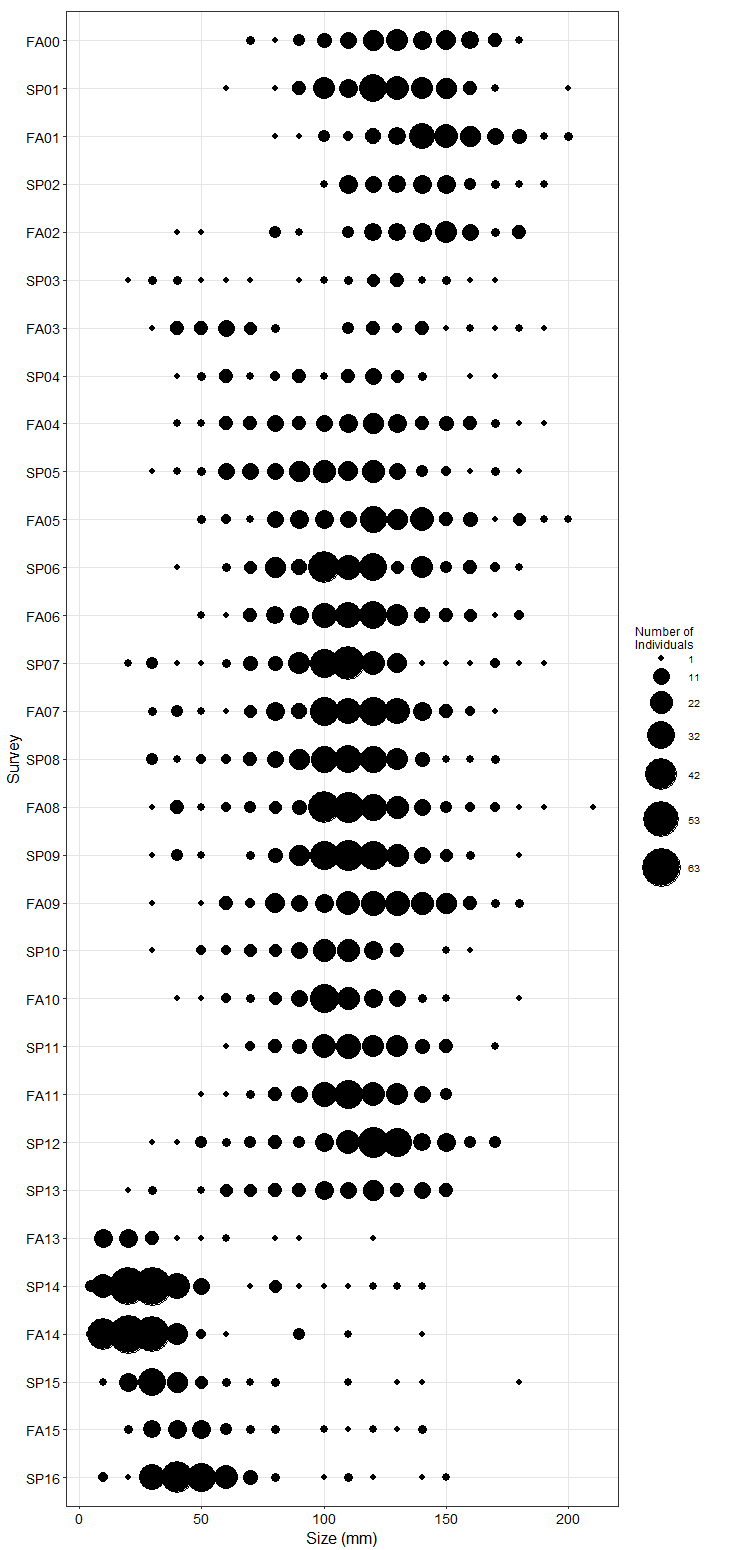

Supplement: S2 Fig — Sizes are radial measurements (see methods) and surveys are labeled as spring (Feb-Apr) or fall (Oct-Nov) samples for a given year. Bubble size represents number of individuals recorded for each size bin. Size frequency graphs can be found for all sites here: http://www.eeb.ucsc.edu/pacificrockyintertidal/sites/sites-target-species.html#pisaster (PNG) [file pone.0192870.s002.png]
