# Supplementary material for: Large-scale impacts of sea star wasting disease (SSWD) on intertidal sea stars and implications for recovery
Source: PLoS One. 2018 Mar 20;13(3):e0192870. doi: 10.1371/journal.pone.0192870 (PMC5860697; doi:10.1371/journal.pone.0192870)
Supplement: S1 Table — Site numbers refer to the maps in S1A–S1H Fig. Monitoring groups include: Sitka Sound Science Center (SSSC), University of California Santa Cruz (UCSC), University of British Columbia (UBC), Olympic National Park (ONP), Padilla Bay National Estuarine Research Reserve (PBNERR), Olympic Coast National Marine Sanctuary (OCNMS), Redwoods National and State Park (RNSP), Point Reyes National Seashore (PORE), Golden Gate National Recreation Area (GOGA), University of California Los Angeles (UCLA), California State University Fullerton (CSUF), California State Polytechnic University Pomona (CPP), Cabrillo National Monument (CABR), Channel Islands National Park (CHIS). (PDF) [file pone.0192870.s003.pdf]

Site No.	Geo Region	Site Name	Latitude	Longitude	Monitoring Group	Pre-2013 Density Data
1	AK	Sage Rock	57.0487	-135.3231	SSSC/UCSC	x
2	AK	Pirates Cove	56.98661	-135.3776	SSSC/UCSC	x
3	BC	Point Atkinson	49.3302	-123.2652	UBC	x
4	BC	W. Vancouver Rope Site	49.3392	-123.2228	UBC	x
5	BC	Ferguson Point	49.306	-123.1568	UBC	x
6	BC	Dunbar	49.2734	-123.1847	UBC	x
7	BC	Yellow Point	49.0431	-123.7509	UBC	x
8	WA Salish Sea	American Camp	48.45731	-123.0222	ONP	
9	WA Salish Sea	Post Point	48.712	-122.5191	UCSC	
10	WA Salish Sea	Saddlebag North Cove	48.5371	-122.5547	PBNERR	
11	WA Salish Sea	Saddlebag South East	48.5343	-122.5549	PBNERR	
12	WA Salish Sea	Hat Island West	48.52353	-122.5522	PBNERR	
13	WA Salish Sea	Hat Island East	48.52221	-122.5435	PBNERR	
14	WA Olympic Coast	Kydikabbit Point	48.39147	-124.6537	OCNMS	
15	WA Olympic Coast	Point of the Arches	48.242	-124.7078	ONP	
16	WA Olympic Coast	Sokol Point	47.94709	-124.6623	ONP	x
17	WA Olympic Coast	Taylor Point	47.86602	-124.57	ONP	x
18	WA Olympic Coast	Starfish Point	47.6527	-124.3905	ONP	x
19	WA Olympic Coast	Point Grenville	47.30255	-124.2753	OCNMS	
20	OR	Ecola	45.91809	-123.9803	ONP/UCSC	x
21	OR	Fogarty Creek	44.83864	-124.0588	UCSC	x
22	OR	Bob Creek	44.24456	-124.1144	UCSC	x
23	OR	Cape Arago	43.30894	-124.4008	UCSC	x
24	OR	Burnt Hill	42.22814	-124.3879	UCSC	x
25	CA North	Enderts	41.69	-124.1426	RNSP	
26	CA North	Damnation Creek	41.65249	-124.1278	RNSP	x
27	CA North	False Klamath Cove	41.59476	-124.1064	RNSP	
28	CA North	Cape Mendocino	40.341	-124.3632	UCSC	x
29	CA North	Shelter Cove	40.02254	-124.0737	UCSC	x
30	CA North	Kibesillah Hill	39.60412	-123.7889	UCSC	x
31	CA North	Sea Ranch	38.7305	-123.4886	UCSC	x
32	CA North	Bodega	38.3182	-123.0737	UCSC	x
33	CA North	Santa Maria Creek	38.01243	-122.8491	PORE	x
34	CA North	Bolinas Point	37.90354	-122.7272	PORE	x
35	CA North	Slide Ranch	37.87406	-122.6009	GOGA	
36	CA North	Point Bonita	37.81911	-122.5293	GOGA	
37	CA Central	Alcatraz	37.825	-122.4219	GOGA	x
38	CA Central	Scott Creek	37.04425	-122.2349	UCSC	x
39	CA Central	Terrace Point	36.94841	-122.0646	UCSC	x
40	CA Central	Hopkins	36.6212	-121.9073	UCSC	x
41	CA Central	Stillwater	36.56087	-121.9405	UCSC	x
42	CA Central	Point Lobos	36.51366	-121.9469	UCSC	x
43	CA Central	Andrew Molera	36.28061	-121.8632	UCSC	x
44	CA Central	Mill Creek	35.97965	-121.4903	UCSC	x
45	CA Central	Point Sierra Nevada	35.72883	-121.3187	UCSC	x

46	CA Central	Cayucos	35.44739	-120.9498	UCSC	x
47	CA Central	Hazards	35.28966	-120.8833	UCSC	x
48	CA Central	Shell Beach	35.16881	-120.6967	UCSC	x
49	CA Central	Occulto	34.88122	-120.6395	UCSC	
50	CA Central	Stairs	34.73038	-120.6155	UCSC	x
51	CA Central	Boat House	34.55388	-120.6117	UCSC	x
52	CA South	Government Point	34.44334	-120.4565	UCSC	x
53	CA South	Alegria	34.46714	-120.2774	UCLA	x
54	CA South	Arroyo Hondo	34.47344	-120.1454	UCLA	x
55	CA South	Coal Oil Point	34.40693	-119.878	UCLA	x
56	CA South	Carpinteria	34.38703	-119.5141	UCLA	x
57	CA South	Mussel Shoals	34.35548	-119.4407	UCLA	x
58	CA South	Old Stairs	34.06612	-118.9978	UCLA	x
59	CA South	Paradise Cove	34.01201	-118.7922	UCLA	x
60	CA South	White Point	33.71539	-118.3196	UCLA	x
61	CA South	Point Fermin	33.70679	-118.286	UCLA	x
62	CA South	Crystal Cove	33.57078	-117.8377	CSUF/CPP	x
63	CA South	Shaws Cove	33.54477	-117.7994	CSUF/CPP	x
64	CA South	Treasure Island	33.51335	-117.7579	CSUF/CPP	
65	CA South	Dana Point	33.4599	-117.7147	CSUF/CPP	x
66	CA South	Cardiff Reef	32.9998398	-117.27867	CABR	x
67	CA South	Scripps Reef	32.8716507	-117.25328	CABR	x
68	CA South	Cabrillo I	32.6694717	-117.2455	CABR	x
69	CA Channel Is. North	Otter Harbor	34.05203	-120.4076	CHIS	
70	CA Channel Is. North	Harris Point	34.07337	-120.3632	CHIS	
71	CA Channel Is. North	Cuyler Harbor	34.04855	-120.3364	CHIS	
72	CA Channel Is. North	Crook Point	34.02159	-120.3789	CHIS	
73	CA Channel Is. North	Fossil Reef	33.99315	-120.2376	CHIS	x
74	CA Channel Is. North	NW Talcott	34.00957	-120.2176	CHIS	x
75	CA Channel Is. North	East Point	33.9427	-119.968	CHIS	x
76	CA Channel Is. North	Ford Point	33.91453	-120.0502	CHIS	x
77	CA Channel Is. North	Johnsons Lee	33.90872	-120.0863	CHIS	x
78	CA Channel Is. North	Trailer	34.05187	-119.9032	CHIS	x
79	CA Channel Is. North	Fraser Cove	34.06265	-119.9192	CHIS	x
80	CA Channel Is. North	Orizaba Cove	34.04508	-119.7219	CHIS	
81	CA Channel Is. North	Prisoners Harbor	34.02038	-119.6867	CHIS	x
82	CA Channel Is. North	Scorpion Rock	34.04552	-119.5469	CHIS	
83	CA Channel Is. North	Willows Anchorage	33.96183	-119.7549	CHIS	x
84	CA Channel Is. North	Cat Rock	34.00557	-119.4193	CHIS	x
85	CA Channel Is. North	Middle West	34.0059	-119.3965	CHIS	x
86	CA Channel Is. North	S Frenchys Cove	34.0066	-119.4108	CHIS	x
87	CA Channel Is. South	Landing Cove	33.48158	-119.0296	CHIS	
88	CA Channel Is. South	Sea Lion Rookery	33.47201	-119.0307	CHIS	x
89	CA Channel Is. South	Bird Rock	33.45145	-118.4876	UCLA	x
90	CA Channel Is. South	Little Harbor	33.38499	-118.4752	UCLA	x
